# Lactoferrin Alleviates Ethanol-Induced Injury via Promoting Nrf2 Nuclear Translocation in BRL-3A Rat Liver Cells

**DOI:** 10.3390/ijms242316848

**Published:** 2023-11-28

**Authors:** Deming Li, Li Ding, Yilin Yan, Yifei Xing, Jiaying Xu, Liqiang Qin

**Affiliations:** 1 School of Public Health, Suzhou Medical College of Soochow University, 199 Renai Road, Suzhou 215123, China; lideming@alu.suda.edu.cn (D.L.); 20224247007@stu.suda.edu.cn (L.D.); yvonne_2468@163.com (Y.Y.); xingyifei1234@163.com (Y.X.); 2 State Key Laboratory of Radiation Medicine and Protection, School of Radiation Medicine and Protection, Suzhou Medical College of Soochow University, 199 Renai Road, Suzhou 215123, China

**Keywords:** lactoferrin, ethanol-induced injury, alleviates, Nrf2, BRL-3A rat liver cell line

## Abstract

Our previous animal studies found that the preventive effects of lactoferrin (Lf) on alcoholic liver injury (ALI) are associated with nuclear factor E2-related factor 2 (Nrf2). To further explore the causality, experiments were performed using rat normal liver BRL-3A cells. Lf treatment reduced ethanol-induced death and apoptosis; meanwhile, Lf treatment alleviated excessive LDH release. These findings confirmed the protection of Lf against ethanol-induced injury in BRL-3A cells. Mechanistically, Lf treatment reversed the reduction in nuclear Nrf2 induced by ethanol without affecting the cytoplasmic Nrf2 level, which led to antioxidant enzyme activity restoration. However, the blocking of Nrf2 nuclear translocation by ML385 eliminated the protective effects of Lf. In a conclusion, Lf protects BRL-3A cells from ethanol-induced injury via promoting Nrf2 nuclear translocation.

## 1. Introduction

Alcoholic liver injury (ALI) is a serious public issue worldwide, and over 50% of cirrhosis-related mortalities are attributable to alcohol consumption [1]. Nutritional intervention plays an important role in ALI managements [2]. Lactoferrin (Lf), mainly derived from bovine milk, is an iron-binding protein with various biological activities [3,4]. Our previous studies have confirmed that Lf can prevent ALI in mice, and the mechanisms are associated with hepatic redox homeostasis regulation [5,6,7]. Nuclear factor E2-related factor 2 (Nrf2) is a central regulator for redox balance, and our animal experiments highlighted the key role of Nrf2 in Lf-mediated ALI prevention [6,7]. Besides our studies, other research groups have also indicated that Lf treatment is linked to Nrf2 activation in different organs or cells [8,9,10,11]. However, the causality may not be determined.

Under unperturbed conditions, Nrf2 is mainly located in the cytoplasm and keeps a relatively low basal level [12,13]. In response to a redox imbalance, Nrf2 is translocated into the nucleus, resulting in antioxidative system up-regulation to maintain redox homeostasis [12,13]. Alcohol intake can lead to Nrf2 gene deregulation, which increases the oxidative stress level and ultimately results in liver injury [14]. Thus, it is a plausible strategy to prevent ethanol-induced liver injury via regulating Nrf2.

Some studies selected HepG2 cells to research ALI [15]; however, due to their lack of expression of key enzymes of ethanol metabolism, especially CYP2E1, most scientists do not think HepG2 cells are suitable for establishing an ALI model [16,17,18]. For other common liver hepatocytes, human normal liver L02 (HL-7702) cells are not collected by an American-type culture collection (ATCC) and have been confirmed to be contaminated with Hela cells [19,20]. Human primary hepatocytes are hard to obtain due to ethical issues. The culture requirements of the immortalized human liver cells THLE-2 and THLE-3 are relatively demanding. A normal mouse liver AML12 cell culture requires the addition of transferrin [21]. Lf belongs to transferrin; thus, the added transferrin may interact or interfere with Lf, resulting in an inaccurate result. Thus, we selected BRL-3A cells to perform this study.

Our understanding of the effects of Lf on Nrf2 in hepatocytes is currently limited. Therefore, this study was conducted to investigate the protective effects of Lf on ethanol-induced hepatocyte injury and explore the causality between Nrf2 and these protective effects.

## 2. Results

### 2.1. The Dosage of Ethanol and Lf

**Figure 1:**
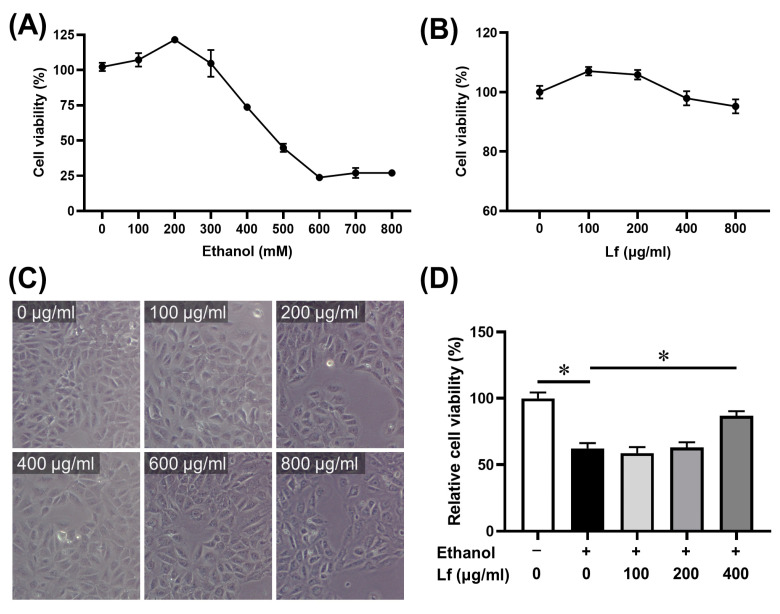
The determination of the doses of Lf and ethanol. (**A**) Effects of ethanol at different doses on cell viability. (**B**) Effects of Lf at different doses on cell viability. (**C**) Effects of different doses of Lf on morphology change. (**D**) Protective effects of Lf against cell viability decrease induced by ethanol (400 mM). N ≥ 6. * *p* < 0.05.

The BRL-3A cell line was used in our experiment. Firstly, we determined the cell viability after being treated with different concentrations of ethanol or Lf for 24 h using the CCK-8 assay. As shown in Figure 1A, for an ethanol concentration within 200 mM, the cell viability was slightly elevated, while the inhibition of cell viability was increased with increasing ethanol doses. The cell viability sharply decreased at the exposure level of 400 mM of ethanol, so 400 mM of ethanol was selected for the subsequent experiments to explore the molecular mechanism. The cell viability and morphology were not obviously affected by Lf at a concentration of 0~800 μg/mL (Figure 1B,C). Our results show that when the BRL-3A cells were treated with both ethanol and Lf and cultured for 24 h, Lf at a concentration of 400 μg/mL significantly attenuated the inhibitory effect of ethanol on the BRL-3A cell viability (Figure 1D).

### 2.2. Effects of Lf on Cell Death and Apoptosis

**Figure 2:**
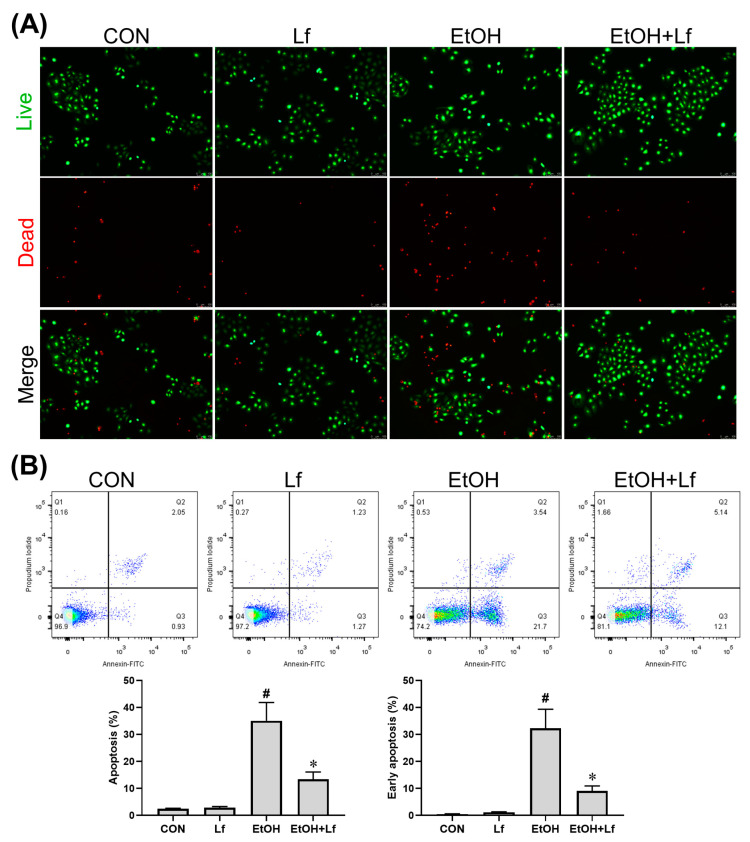
Effects of Lf (400 μg/mL) and ethanol (400 mM) on death and apoptosis of BRL-3A cells. (**A**) Effect of ethanol and Lf on cell death. (**B**) Effect of ethanol and Lf on apoptosis. N = 3. EtOH vs. CON, ^#^ *p* < 0.05; EtOH+Lf vs. EtOH, * *p* < 0.05.

As shown in Figure 2A, compared with the control (CON) and Lf groups, the number of dead cells was obviously increased in the ethanol stimulation (EtOH) group, which was alleviated by the Lf treatment. Similarly, Lf could reduce ethanol-induced cell apoptosis, especially early apoptosis (Figure 2B).

### 2.3. Protective Effects of Lf on Ethanol-Induced Injury in BRL-3A Cells

**Figure 3:**
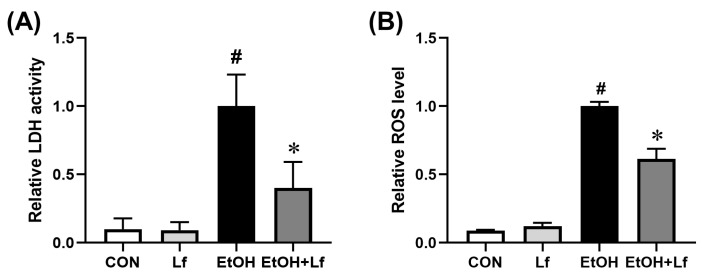
Protective effects of Lf on ethanol-induced injury in BRL-3A cells. (**A**) Effect of Lf on LDH activity level of cell culture supernatant. (**B**) Effect of Lf on intracellular ROS level. N = 6. EtOH vs. CON, ^#^ *p* < 0.05; EtOH+Lf vs. EtOH, * *p* < 0.05.

The lactate dehydrogenase (LDH) activity level in a cell culture supernatant is a classical biomarker for cytotoxicity. As shown in Figure 3A, compared with the CON group, the LDH activity of the culture medium increased by approximately 10 times in the EtOH group; meanwhile, the Lf treatment reduced the ethanol-induced LDH activity by nearly 60% (*p* = 0.014). The ethanol stimulation induced a 10-fold increase in the intracellular reactive oxygen species (ROS) level, which could be reduced by half with the Lf treatment (*p* < 0.01) (Figure 3B).

### 2.4. Effects of Lf on Cytoplasmic and Nuclear Protein Expression in BRL-3A Cells

**Figure 4:**
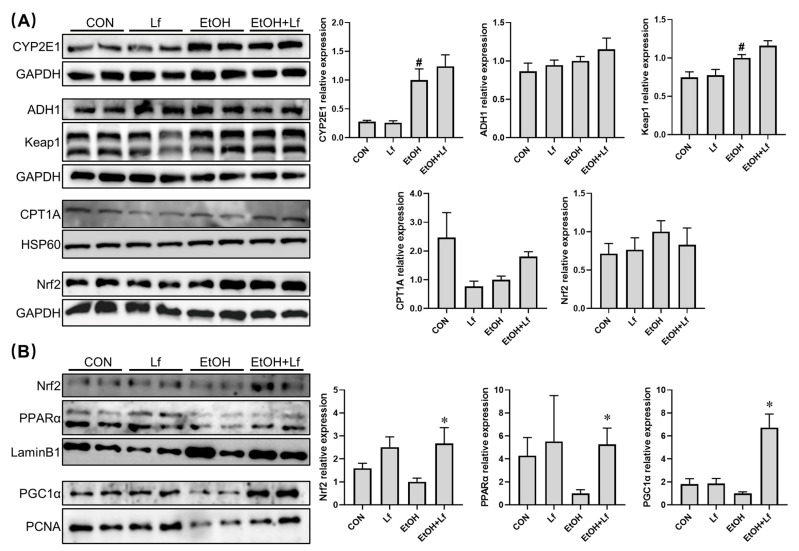
Effect of Lf on the expression of proteins in BRL-3A cells. (**A**) Cytoplasmic protein expression levels in BRL-3A cells. (**B**) Nuclear protein expression levels in BRL-3A cells. N ≥ 4. EtOH vs. CON, ^#^ *p* < 0.05; EtOH+Lf vs. EtOH, * *p* < 0.05.

CYP2E1 and ADH1 are the key enzymes for alcohol metabolism [22]; Keap1 and Nrf2 play an important role in redox sensation and regulation [13]; PPARα and PGC1α participate in fatty acid β-oxidation [23]. All the above signaling pathways are closely associated with ALI. Thus, we tested their protein expression levels with Western blots. As shown in Figure 4A, the ethanol stimulation induced a four-fold CYP2E1 protein overexpression in the BRL-3A cells (*p* = 0.003) and up-regulated the cytoplasmic Keap1 protein level by about one-third (*p* = 0.016). Although without statistical significance, compared with the CON group, CPT1A expression was decreased in the EtOH group, while it could be nearly doubled with the Lf treatment. Meanwhile, there was no difference in alcohol dehydrogenase 1(ADH1) or Nrf2 protein levels among the four groups.

Further, we determined the nuclear protein expression levels (Figure 4B). Compared with the CON group, although the differences were insignificant, the ethanol stimulation tended to decrease the nuclear protein expressions of Nrf2, PPARα, and PGC1α. Compared with the EtOH group, in the EtOH+Lf group, the Nrf2, PPARα, and PGC1α nuclear protein levels were up-regulated by about 1.5 fold (*p* = 0.01), 4 fold (*p* = 0.03), and 5.5 fold (*p* < 0.01), respectively.

### 2.5. Effects of Lf on AntiOxidant Enzyme Activities

**Figure 5:**
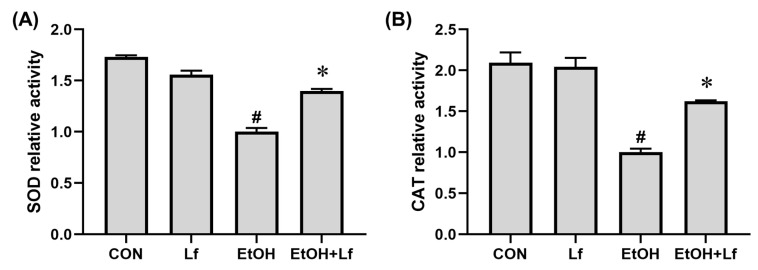
Effects of Lf on antioxidant enzyme activities. (**A**) Effect of Lf on SOD activity level. (**B**) Effect of Lf on CAT activity level. N = 3. EtOH vs. CON, ^#^ *p* < 0.05; EtOH+Lf vs. EtOH, * *p* < 0.05.

As shown in Figure 5, compared with the CON group, the Lf treatment alone did not affect the superoxide dismutase (SOD) and catalase (CAT) activities; however, the ethanol stimulation reduced the SOD and CAT activities by 40% (*p* < 0.01) and 54% (*p* < 0.01), respectively. Meanwhile, compared with the EtOH group, the SOD and CAT activities increased by about 40% (*p* < 0.01) and 60% (*p* < 0.01) in the EtOH+Lf group, respectively.

### 2.6. Effects of ML385 on the Protective Role of Lf in Ethanol-Induced Cell Injury

**Figure 6:**
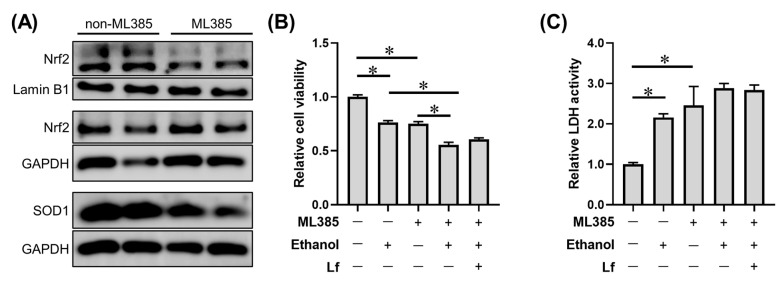
Effects of ML385 on the protective role of Lf in ethanol-induced cell injury. (**A**) Effects of ML385 on protein expressions in BRL-3A cells. (**B**) Effects of Lf and ethanol on cell viability in the presence of ML385. (**C**) Effects of ML385 on the LDH activity of the cell culture supernatant in the presence of ML385. The dose of ML385 is 2 µM. N ≥ 4. * *p* < 0.05.

To further explore the role of Nrf2 nuclear translocation in the protection of Lf against ethanol, ML385, a small molecular antagonist of Nrf2 nuclear translocation [24,25], was applied in our experiment. As shown in Figure 6A, ML385 significantly reduced the nuclear Nrf2 protein level without affecting the cytoplasmic Nrf2 content; meanwhile, SOD1, a Nrf2 downstream protein, was down-regulated by the ML385 application. ML385 exacerbated the reduction in cell viability induced by the ethanol stimulation; meanwhile, we observed that Lf lost its protective effect on cell viability in the presence of ML385 (Figure 6B). Even with the ML385 intervention, the Lf treatment exacerbated the ethanol-induced LDH release in BRL-3A cells (Figure 6C).

## 3. Discussion

In this study, using rat normal liver BRL-3A cells as the experimental subject, we found that Lf protects cell injuries induced by ethanol via promoting Nrf2 nuclear translocation.

BRL-3A cells have been collected by an ATCC and used to conduct ALI studies by other experts [26,27]. Thus, we selected BRL-3A cells to perform this study. In the pre-culture stage, we found that DMEM with 5% FBS can maintain the optimal growth and proliferation of BRL-3A cells. Thus, the FBS concentration of the complete medium was set at 5% instead of the most common 10%.

According to previous studies [26,27] and our CCK-8 assay (Figure 1), the ethanol stimulation dose was set at 400 mM. Because 400 mM of ethanol induced a mild injury in the BRL-3A cells, which was possibly prevented by the Lf treatment, it might be hard for Lf, as a natural product [28], to alleviate more severe injuries induced by higher doses of ethanol. Lf at a concentration of 400 μg/mL not only did not induce cytotoxicity but also rescued the ethanol-induced cell viability decrease (Figure 1). Thus, we selected this dose as the Lf treatment dose. The Lf treatment alleviated the cell death and apoptosis (especially early apoptosis) induced by the ethanol stimulation (Figure 2). Further, the Lf treatment could reduce ethanol-induced cytotoxicity and intracellular ROS accumulation (Figure 3). Similar to the animal experiments [5,6,7], Lf also exhibited preventive effects on ALI in vitro.

In the liver, ADH and CYP2E1 are the key enzymes for alcohol metabolism, and ADH1 is the most important ADH subtype [22]. Ethanol exposure can induce CYP2E1 overexpression [29], which was further confirmed in our present study. However, compared with the EtOH group, the protein expression levels of ADH1 and CYP2E1 were no different in the EtOH+Lf group (Figure 4A). These results suggested that Lf does not affect alcohol metabolism. Our previous studies indicated that Lf may improve fatty acid β-oxidation to prevent ALI [5,6,7]. The present study also found that Lf tended to up-regulate the protein expression of CPT1A, the fatty acid β-oxidation key enzyme (Figure 4A). Additionally, intranuclear PPAR-α and PGC1α play a critical role in fatty acid β-oxidation, and PPAR-α activation is beneficial to ALI alleviation [23]. We found that Lf up-regulates PPAR-α and PGC1α protein expressions in the presence of ethanol (Figure 4B). These results further confirmed that Lf can improve ethanol-induced fatty acid β-oxidation abnormalities.

**Figure 7:**
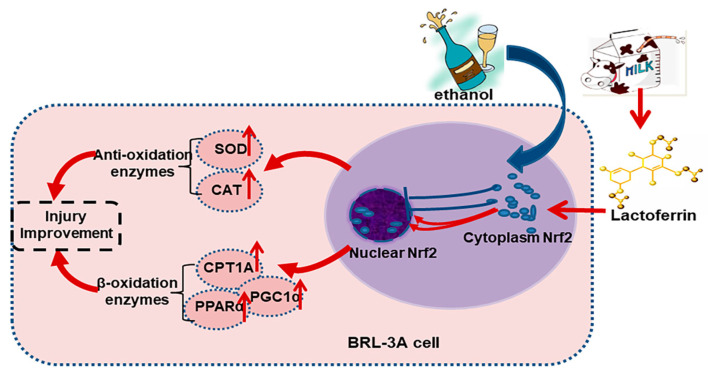
The potential mechanisms for the preventive effects of Lf on ethanol-induced cell injury.

The Keap1-Nrf2 signaling pathway is a central regulator of redox homeostasis [13]. Our animal experiments also emphasized the importance of Nrf2 in the preventive effects of Lf on ALI [6,7]. In the cytoplasm, although Lf did not change the Nrf2 protein level, the ethanol simulation significantly increased Keap1 expression, a negative regulator of Nrf2. Nrf2 in the cytoplasm is usually inactive due to Keap1-mediated continuous ubiquitylation and degradation, and Nrf2 produces biological activity after translocating into the nucleus [13]. Other researchers found that Lf treatment can up-regulate the Nrf2 expression protein level; however, the sub-cellular localization of the Nrf2 protein was not explored [10,11,30]. Thus, we further determined the nuclear Nrf2 protein expression. Lf can restore the nuclear Nrf2 expression decrease induced by ethanol exposure (Figure 4B). Nrf2 is responsible for the regulation of antioxidation enzymes [13]. In the present study, SOD and CAT activities are positively related to the nuclear Nrf2 level (Figure 5), which may explain why the ROS levels in the EtOH+Lf group are lower than those in the EtOH group. An Nrf2 translocation inhibitor was used to verify the causality between the promotion of Nrf2 nuclear translocation by Lf and Lf’s preventive effects on ALI. ML385 is a specific small-molecule Nrf2 translocation inhibitor [24]. In this study, ML385 decreased the Nrf2 level in the nucleus without affecting the Nrf2 content in the cytoplasm; meanwhile, the protein expression of the Nrf2 downstream protein SOD1 was suppressed by ML385 (Figure 6A). These results confirmed the antagonism of ML385 on Nrf2 nuclear translocation. In the presence of ML385, Lf lost its protective effects on enhancing cell viability and decreasing LDH release and even exacerbated the ethanol-induced cellular LDH release (Figure 6B,C). This can be inferred from the evidence that Lf exerts a protective effect against ethanol-induced cellular injury through promoting Nrf2 nuclear translocation. The potential mechanisms are exhibited in Figure 7. Analogous findings were also reported in the nervous system [31]. Zakharova et al. indicated that iron-free Lf is able to induce the translocation of Nrf2 from the cytoplasm to the nucleus [31].

Our study has some limitations. On the one hand, only BRL-3A cells were included in the present study. The generality of our conclusion needs further confirming using other cell lines. On the other hand, Nrf2 transgenic mice should be applied in a future study, which is helpful to illustrate the role of Nrf2 at the animal level.

## 4. Materials and Methods

### 4.1. Cells and Reagents

Rat normal liver BRL-3A cells were purchased from Fuheng Biology (Shanghai, China). Bovine Lf (14% iron saturation) was purchased from Hilmar Cheese (Delhi, CA, USA). Anhydrous ethanol (guaranteed reagent) was purchased from Chinasun Specialty Products Co., Ltd. (Suzhou, China). Dulbecco’s modified Eagle’s medium (DMEM) (high glucose; non-phenol red and non-sodium pyruvate) and fetal bovine serum (FBS) were purchased from Vivacell (Shanghai, China). ML385 was purchased from Selleck (Shanghai, China). A CCK-8 kit, protein-loading buffer, and ECL reagent were purchased from FudeBio (Hangzhou, China). A lactate dehydrogenase (LDH) release assay kit and total SOD assay kit were purchased from Beyotime (Shanghai, China). A calein-AM/PI double-staining (live/dead) assay kit was purchased from Yeasen (Shanghai, China). An apoptosis detection kit was purchased from Bioworld (Nanjing, China). A catalase (CAT) activity assay kit was purchased from Solarbio (Beijing, China). An ROS assay kit was purchased from Donjindo (Shanghai, China). A nuclear and cytoplasmic protein extraction kit was purchased from FudeBio (Hangzhou, China). The antibody of CYP2E1 (1/2000) was purchased from Abcam (Cambridge, UK). The antibody of ADH1 (1/1000) was purchased from CST (Danvers, MA, USA). The antibodies of GAPDH (1/20000), Keap1 (1/5000), CPT1A (1/2000), HSP60 (1/2000), Nrf2 (1/3000), and Lamin B1 (1/2000) were purchased from Proteintech (Wuhan, China). The antibodies of PPARα (1/1000), PGC1α (1/2000), PCNA (1/2000), and SOD1 (1/1000) were purchased from ABclonal (Wuhan, China).

### 4.2. Cell Culture

BRL-3A cells were cultured in DMEM supplemented with 5% FBS at 37 °C in 5% CO_2_. The cell culture and passage were conducted according to the common protocols [32].

### 4.3. Measurement of Cell Viability

The CCK-8 assay was performed to test the cell viability. Briefly, BRL-3A cells were seeded in a 96-well plate at a density of 5000 cells per well. After cell adherence, the medium was replaced with a fresh medium containing ethanol or Lf. After incubation for 24 h, the medium was replaced with 100 μL of a medium, mixing 10 μL of CCK-8 assay solutions. After incubation for 2 h, the optical density (OD) value of each well was measured at 450 nm. The cell viabilities were calculated according to “Cell viability = [(OD_treatment_ − OD_blank_)/(OD_control_ − OD_blank_)] × 100%”.

### 4.4. Measurement of Cytotoxicity

The leakage of LDH is a cytotoxicity biomarker [33]. The LDH activity of the medium was determined according to the manufacturer’s instructions.

### 4.5. Cell Treatment

BRL-3A cells were divided into four groups after cell attachment for 24 h and received the following treatments for another 24 h:

1. Control (CON) group: complete medium.
2. Lf group: complete medium containing 400 μg/mL of Lf.
3. Ethanol model (EtOH) group: complete medium containing 400 mM of ethanol.
4. Lf+EtOH group: complete medium containing 400 mM of ethanol and 400 μg/mL of Lf.

### 4.6. Measurement of Live/Dead Cells

After the treatments, the culture medium was removed, and the cells were washed using the assay buffers. Then, the assay buffers, mixed with Calcein-AM and propidium iodide (PI), were added. The cells were incubated at 37 °C for 30 min. Images of the live (green) and dead (red) cells were observed and obtained using an inverted fluorescence microscope.

### 4.7. Measurement of Cell Apoptosis

The suspension cells were collected by centrifugating them at 1000 rpm. The cells were washed using pre-cold PBS twice. A binding buffer containing Annexin V-FITC and PI was added to the cell suspensions. After mixing gently, the cells were incubated at 4 °C for 10 min in a light-avoiding environment. Finally, the apoptosis was analyzed using flow cytometry within 1 h.

### 4.8. Measurements of Intracellular ROS Level and Antioxidant Enzyme Activity

The cells were seeded in a black 96-well plate. After the treatments, the intracellular ROS levels were determined using the ROS assay kit with a fluorescence microplate reader. Then, the treated cells cultured in 100 mm culture dishes were harvested, adjusted to 8 million cells per sample, and collected by centrifugating them at 1000 rpm. The SOD and CAT activities were determined using the corresponding kits according to the instructions.

### 4.9. Western Blots

The nuclear and cytoplasmic proteins were separately extracted. Briefly, the cells were collected after washing with ice-cold PBS and centrifugation at 4 °C. The cell sediments were dissolved with cytoplasmic protein extraction agent A. After violent vortexing and incubation on ice, cytoplasmic protein extraction agent B was added. After vortexing and incubation on ice, the samples were centrifuged for 5 min at 12,000× *g*, and then the supernatants (cytoplasmic protein) were obtained. Subsequently, the sediments were dissolved with a nuclear protein extraction agent. After full vortex and lysis, the supernatants (nuclear proteins) were obtained via 12,000× *g* centrifugation for 10 min at 4 °C. Then, they were mixed with a loading buffer and boiled at 98 °C. The remaining Western blot procedures were performed as previously described [34,35]. GAPDH or HSP60 was used as a loading control for the cytoplasmic protein; Lamin B1 or PCNA was used as a loading control for the nuclear protein.

### 4.10. Statistical Analysis

All data were presented as “mean ± standard error (SE)”. A one-way ANOVA followed by an LSD post hoc test was used to compare the differences among the groups. *p* < 0.05 was considered statistically significant. All analyses were performed using SPSS version 21.0 (IBM, New York, NY, USA). Figures were plotted using GraphPad Prism 8.0 (GraphPad Software Inc., San Diego, CA, USA).

## 5. Conclusions

All in all, this study found that Lf alleviates ethanol-induced cell injury via promoting Nrf2 nuclear translocation in BRL-3A rat liver cells.

## Data Availability

The data presented in this study are available on request from the corresponding author.
